# The oxytocin/vasopressin receptor antagonist atosiban delays the gastric emptying of a semisolid meal compared to saline in human

**DOI:** 10.1186/1471-230X-6-11

**Published:** 2006-03-16

**Authors:** Bodil Ohlsson, Ola Björgell, Olle Ekberg, Gassan Darwiche

**Affiliations:** 1 Departments of Clinical Sciences, Entrance 35, S-205 02 Malmö, Sweden; 2 Diagnostic Radiology, Malmö, Lund University, Sweden

## Abstract

**Background:**

Oxytocin is released in response to a meal. Further, mRNA for oxytocin and its receptor have been found throughout the gastrointestinal (GI) tract. The aim of this study was therefore to examine whether oxytocin, or the receptor antagonist atosiban, influence the gastric emptying.

**Methods:**

Ten healthy volunteers (five men) were examined regarding gastric emptying at three different occasions: once during oxytocin stimulation using a pharmacological dose; once during blockage of the oxytocin receptors (which also blocks the vasopressin receptors) and thereby inhibiting physiological doses of oxytocin; and once during saline infusion.

Gastric emptying rate (GER) was assessed and expressed as the percentage reduction in antral cross-sectional area from 15 to 90 min after ingestion of rice pudding. The assessment was performed by real-time ultrasonography. At the same time, the feeling of satiety was registered using visual satiety scores.

**Results:**

Inhibition of the binding of endogenous oxytocin by the receptor antagonist delayed the GER by 37 % compared to saline (p = 0.037). In contrast, infusion of oxytocin in a dosage of 40 mU/min did not affect the GER (p = 0.610). Satiation scores areas in healthy subjects after receiving atosiban or oxytocin did not show any significant differences.

**Conclusion:**

Oxytocin and/or vasopressin seem to be regulators of gastric emptying during physiological conditions, since the receptor antagonist atosiban delayed the GER. However, the actual pharmacological dose of oxytocin in this study had no effect. The effect of oxytocin and vasopressin on GI motility has to be further evaluated.

## Background

During the last years it has been suggested that oxytocin contribute to the control of the gastrointestinal (GI) motility. Oxytocin is secreted into the blood in response to endogenous stimulation with a fatty meal, and exogenous stimulation with cholecystokinin (CCK) [1]. Both mRNA for oxytocin and its receptor have been found throughout the human GI tract [2]. Oxytocin has also been shown to accelerate gastric emptying of a meal in healthy individuals [3], and has been used to treat prolonged post-vagotomy gastric atony [4]. Further, oxytocin has been shown to stimulate colonic peristalsis [5].

The effects observed throughout the GI tract suggest that oxytocin plays a critical role in GI motility. The aim of this study was to further examine the effect of oxytocin on gastric emptying in healthy individuals, by examining the gastric emptying rate (GER) during continuous infusion of either oxytocin or the oxytocin receptor antagonist atosiban.

## Methods

The study was performed according to the Helsinki declaration and approved by the Ethics Committee at Lund University. All subjects gave written, informed consent before the experiments.

### Subjects

Ten healthy volunteers (5 women) with a mean age of 40 ± 16 years (range, 25–62 years) and a body mass index of 23.3 ± 1.7 kg/m^2 ^(range, 20.8 – 26.7 kg/m^2^), without symptoms or a prior history of GI disease, abdominal surgery or diabetes mellitus were studied. None of the subjects were using any pharmaceutical drugs affecting gut motility. All subjects were recruited from the population of a southern county of Sweden. Two of the subjects were smokers. They all underwent a basal physical examination before inclusion in the study.

### Test Meal

Each subject was given 300 g of rice pudding (Scan Risgrynsgröt; Scan Foods, Johanneshov, Sweden). The total energy value for the rice pudding was 1386 kJ, provided as 10 % protein, 58 % carbohydrate, and 32 % fat. The nutrient composition per 100 g rice pudding was 3 g protein, 16 g carbohydrate and 4 g fat.

### Drugs

The subjects were randomly examined at three different occasions given either infusion of saline, oxytocin or an oxytocin receptor antagonist. One ml of Syntocinon^® ^(Novartis, Täby, Sweden), a synthetic analogue to oxytocin, with a concentration of 5 U/ml was dissolved in 250 ml NaCl and given as an intravenous infusion at a rate of 2 ml/min, i.e. a dose of 40 mU/min. Tractocile^® ^(atosiban) (Ferring, Malmö, Sweden) is an antagonist to the oxytocin receptor. It was given as an intravenous bolus dose of 6.75 mg atosiban in a concentration of 7.5 mg/ml, immediately followed by a continuous intravenous infusion of 5 ml atosiban in a concentration of 7.5 mg/ml dissolved in 250 ml NaCl and given at a rate of 2 ml/min. This gives a dose of 300 μg atosiban/min. In a third experiment, 2 ml/min of 0.9 % saline was given as an intravenous infusion. The infusions continued for 90 min.

The reason for choosing this dosage of oxytocin was that this has been shown to rise the plasma oxytocin levels remarkable in man (from 2–3 pg/ml to around 50 pg/ml) [6], and it is the highest dosage recommended by the drug company. This dose has been used to treat gastric atony [4]. Further, in an earlier study a dose-response curve was performed, and this dosage had an equal effect compared to 20 mU/min in regard to stimulating colonic peristalsis [5]. The rationale for choosing the atosiban dosage was that this is the dosage recommended for clinical use by the drug company. Although developed as an oxytocin receptor antagonist, it has similar affinity for vasopressin receptors [7,8].

### Ultrasonography

Ultrasonographic measurements of gastric emptying were assessed by a standardised method previously described [9]. The patients were examined with a 3.5-MHz abdominal transducer and an Acuson 128XP 10 system (Siemens Medical Solutions, Mountain View, CA). Gastric emptying was monitored indirectly by determining the longitudinal and anteroposterior diameters of a single section of the gastric antrum, using the abdominal aorta and the left lobe of the liver as internal landmarks to obtain the same standardized scanning level consistently. At each observation, 3 measurements were done with the use of the mean values of the longitudinal (d1) and the anteroposterior (d2) diameters to calculate the cross sectional area of the gastric antrum using the following formula:

Antrum area = π × r^2 ^= π × d1/2 × d2/2 = π × d1 × d2/4

Gastric emptying rate (GER) was assessed and expressed as the percentage reduction in antral cross-sectional area from 15 to 90 min after meal ingestion using the following formula:

GER = [1 – (Antrum area 90 min/Antrum area 15 min)] × 100. This method has been evaluated in comparison to scintigraphic measurements of gastric emptying, and ultrasonographic gastric emptying rate has been shown to strongly correlate to scintigraphic half-time values [10].

### Experimental design

The subjects were examined between 8 and 11 AM, after an 8-hour fast. Smoking was prohibited for 8 h before and during the test. Each subject was checked for normal fasting blood glucose concentration on the day of the examination. If the subjects on the study day reported symptoms from the gastrointestinal tract (i.e. diarrhoea or constipation), the examination was postponed. The test meal had to be ingested within 5 minutes. Gastric emptying was examined ultrasonographically after 15 and 90 minutes and at the same time, as well as prior the study, the subjects had to complete a questionnaire about their grade of hunger and/or feeling of satiety using visual satiety scores with a scoring graded from 0, from extreme hunger, to 20, for extreme satiety. The drug infusion was started at the same time as the start of the meal ingestion, and lasted throughout the experiment. With at least two days in between all subjects underwent the examination three times at different occasions given either infusion of the drugs previously described. The sequence of experiments was randomly assigned and the ultrasound examinations performed by the same radiologist, who was blinded with regard to the drugs given to the subjects.

### Statistical analyses

Values are given as median and interquartile ranges (IQR). The areas under the curves (AUCs) for each subject were determined for the satiety scores (Graph Pad PRISM, San Diego). The areas, GER, the satiety AUCs and the satiety scores between saline and oxytocin respective atosiban were compared using Wilcoxon signed rank test. P < 0.05 was considered statistically significant.

## Results

### Gastric emptying rate (GER)

**Figure 1:**
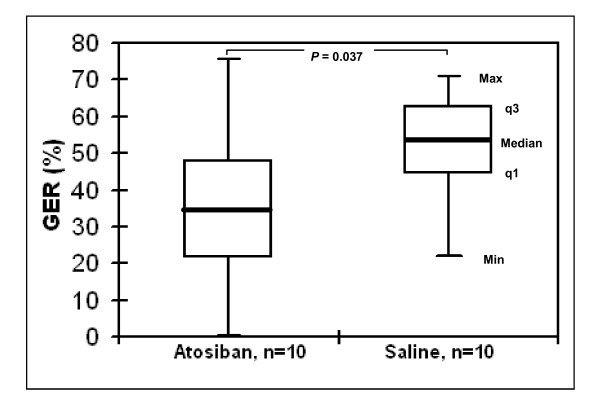
The gastric emptying rate (GER) was decreased in the group receiving atosiban compared to the group receiving saline. Wilcoxon signed rank test.

**Figure 2:**
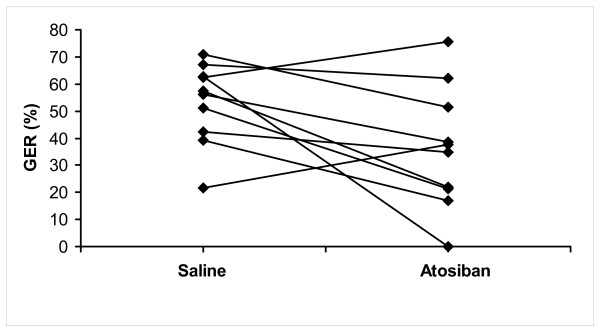
The individual gastric emptying rate (GER) during saline and atosiban. All but two of subjects had a decreased GER during atosiban administration.

The gastric antral area was reduced at 15 min in the group receiving atosiban compared to saline (Table 1). Infusion of saline led to a GER of 57 (44–63) %. During infusion of the oxytocin receptor antagonist atosiban, the GER was significantly reduced by 37 % to 36 (22–48) % (Fig 1). The individual change in GER is shown in Figure 2. In contrast, infusion of oxytocin led to a GER of 44 (39–62) % which did not differ from saline (p = 0.610). Five individuals had a reduced, and 5 had an increased GER during oxytocin infusion compared to saline.

### Satiety

**Figure 3:**
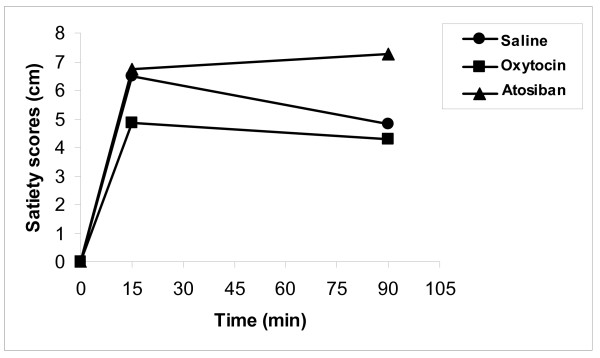
No significant difference could be shown between the relative satiety scores in healthy subjects after receiving oxytocin and atosiban compared to saline, *n *= 10. Satiety score at the start of the experiment was set to 0. Wilcoxon signed rank test.

The AUC at 0–15 min and 0–90 min for satiety was not significantly prolonged after receiving atosiban or oxytocin compared to saline (Table 2). Neither were there any significant differences between the satiety scores (Figure 3).

## Discussion

This study showed a decreased gastric emptying rate after blockage of the oxytocin receptors, whereas a pharmacological dosage of oxytocin did not affect the emptying rate. We have earlier described how oxytocin is released in response to a fatty meal in healthy women (1.0 ± 0.17 basally compared to 1.3 ± 0.26 pmol/l postprandially, p = 0.02) [1] and in patients with diabetes mellitus of both sexes (208.1 ± 148.7 basally compared to 250.6 ± 166.9 pg/ml postprandially, p = 0.02) [Ohlsson et al, unpublished observation], why it can be assumed that oxytocin release is induced also after intake of the present meal. By inhibition of the binding of endogenous oxytocin to the oxytocin receptors, the gastric emptying was delayed. As the antral area was reduced at 15 min after atosiban, one may speculate if retention of food in the proximal ventricle may be partly responsible for the delayed emptying rate, or a rapid initial emptying has occurred. The transport of food from the proximal to the distal part of the ventricle may be as important as the transport from the antrum to the duodenum.

The neurophyseal hormones vasopressin and oxytocin are cyclic nonapetides whose actions are mediated by stimulation of specific G protein-coupled receptors currently classified into V_1 _– vascular, V_2 _– renal and V_3 _– pituitary vasopressin receptors and oxytocin receptors. Vasopressin is the ligand having the highest affinity for the human V-receptors, while oxytocin is the ligand with the highest affinity for the oxytocin receptor. However, there is cross-reactivity of the ligands to the receptors [11].

Atosiban is an analogue of oxytocin and has been rationally designed to compete with endogenous oxytocin at myometrial and decidual oxytocin receptors. Clinical studies have revealed that atosiban is an effective and safe tocolytic agent [12]. However, atosiban has an equal, if not a greater, affinity for vasopressin receptors compared with oxytocin receptors due to their close chemical homology [7,8]. Furthermore, an additional intracellular process may be attributed as atosiban has been shown to dose-dependently inhibit oxytocin-induced second messengers [13,14]. This intracellular inhibition was stronger against oxytocin than against vasopressin [15]. No pure oxytocin receptor antagonist is available for clinical use, and may be very difficult to develop.

Thus, we cannot know that the inhibitory effect of atosiban on the gastric emptying is mediated exclusively through oxytocin receptors. The effects observed due to inhibition of endogenous oxytocin to oxyocin/vasopressin receptors by atosiban may theoretically be due to inhibition of endogenous vasopressin as well. However, the effect of vasopressin on the GI tract is only rudimentary examined, and we do not know if vasopressin is released in response to a meal. The expression of vasopressin receptors in the human GI tract has never been studied to our knowledge, although studies have shown the expression of vasopressin in gastric and duodenal cells in the rat [16]. Vasopressin has been shown to influence gastric motility in women [17]. One study [18] has shown that vasopressin increases the colonic peristalsis in a way similar to oxytocin [5]. The precise mechanism for the action of atosiban in the GI tract has thus to be further evaluated. It remains to settle to what extent oxytocin, and to what extent vasopressin is involved in the regulation of GI motility. In the obstetrics, where atosiban has been developed as a tocolytic drug, this is not a problem as an increased expression of oxytocin receptors but not vasopressin receptors is found in the uterus during labour [19].

Earlier studies in man have shown that oxytocin improves gastric emptying [3,4]. However, oxytocin in our present study failed to improve the emptying rate. This may depend on the dosage of the peptide. The reason for choosing this dosage was that this was the highest dosage recommended by the drug company. Further, this dosage has in an earlier dose-response study been shown to stimulate colonic peristalsis [5]. It is a well-known phenomenon that stimulation of a receptor in increasing dosages may show a bell-shaped response [20,21], explaining divergent effects of the same dosage depending on the receptor affinity and/or amount at different sites of the GI tract. Oxytocin may be of importance for the gastric emptying during physiological conditions, although not our actual pharmacological dosage was. The test meal *per se *may have given a high enough endogenous oxytocin secretion. Stimulation by our modest pharmacological dosage of oxytocin during these circumstances may be of no further benefit. Furthermore, the oxytocin receptor mRNA found [2] may be involved in regulation of slow waves, mixing movements and liquid emptying, effects not possible to be detected by this method. The actual method measured the gastric volume at two different time points, and thereby could the gastric emptying rate be calculated, but the gastric emptying process is not studied in detail.

Petring [3] used dosages of 0.33 U oxytocin/min in altogether 30 min, which should be compared to our dosage of 40 mU oxytocin/min. The dosages used by Hashmonai et al [4] to treat gastric atony were in the range of 20–80 mU/min, but lasted for three days. Although oxytocin has an effect on dysmotility in these dosages on an empty stomach, it is not clear that it might have any effects with the same dosages in healthy volunteers after a meal.

In contrast to the above findings in human, the motility in the rat stomach was inhibited by oxytocin [21,22]. We haven't been able to identify any oxytocin receptors in the rat GI tract (Ohlsson et al, unpublished observation). This might explain why the effects evoked by oxytocin on gastric and intestinal motility in rat are mediated by release of cholcystokinin (CCK) (which inhibits gastric emptying) and CCK receptors, and differ from oxytocin effects evoked in human [21,22].

This study has some limitations. Only one dosage of oxytocin was examined. This was a pilote trial, and it is difficult to examine the same subjects more than three times. Before planning next study, the optimal dosage of oxytocin for gastric motility must be titrated. So far, the interest for vasopressin has been modest, but after the present results also vasopressin will be further evaluated. However, before examining the effect of vasopressin on human GI tract, we have to examine if the receptors are present, and if there is a postprandial vasopressin response.

## Conclusion

Blockage of oxytocin and vasopressin receptors in man inhibited the gastric emptying. It remains to determine whether it is oxytocin or vasopressin, or both, that is most important for gastric motility. However, one or both of these peptides seem to be regulators of gastrointestinal physiology in healthy subjects. The level of action needs to be determined. Further, the role of these peptides in the pathophysiology of gastro paresis remains to be settled.

## Competing interests

The author(s) declare that they have no competing interests.

## Authors' contributions

BO participated in the design of the study, reqruited subjects, paid the study and drafted the manuscript. OB participated in the design of the study, reqruited subjects for the study and performed the ultrasound examinations. OE participated in the design of the study and participated in drafting of the manuscript. GD participated in the design of the study, reqruited subjects, performed the statistical calculations and the graphs and participated in drafting of the manuscript. All authors read and approved the final manuscript.

## Pre-publication history

The pre-publication history for this paper can be accessed here:



## Figures and Tables

**Table 1:** The gastric antral area

Antrum Area	Saline	Oxytocin	Atosiban
	*Cm^2^*	*Cm^2^*	*Cm^2^*

15 min	704 (606 – 796)	622 (420 – 872)	512 (389 – 748)*
90 min	293 (222 – 505)	349 (141 – 522)	315 (230 – 520)

The area between saline and atosiban differed at 15 min, * = p < 0.05. Median, q1-q3; *n *= 10. Wilcoxon signed rank test.

**Table 2:** Satiation scores areas in healthy subjects

Area under the curve	Saline	Oxytocin	Atosiban
	*cm × min*	*cm × min*	*cm × min*

0 – 15 min	934 (736 – 1077)	924 (665 – 1022)	1025 (706 – 1189)
0 – 90 min	118 (90 – 152)	132 (76 – 152)	114 (84 – 152)

Satiation scores areas after receiving oxytocin and atosiban did not differ significantly from saline. Median, q1-q3; *n *= 10. Wilcoxon signed rank test.
